# Body Mass Index and Anthropometric Criteria to Assess Obesity

**DOI:** 10.1001/jamanetworkopen.2025.49124

**Published:** 2025-12-29

**Authors:** Nora M. Al-Roub, Devesh Malik, Mohammed Essa, Yuan Lu, Huanhuan Yang, Erica S. Spatz, Harlan M. Krumholz, Kamil F. Faridi

**Affiliations:** 1 Yale School of Public Health, New Haven, Connecticut; 2 Yale School of Medicine, New Haven, Connecticut; 3 Harvard T.H. Chan School of Public Health, Boston, Massachusetts; 4 Richard A. and Susan F. Smith Center for Outcomes Research, Beth Israel Deaconess Medical Center, Boston, Massachusetts; 5 Section of Cardiovascular Medicine, Department of Medicine, Yale School of Medicine, New Haven, Connecticut; 6 Center for Outcomes Research and Evaluation, Yale New Haven Health, New Haven, Connecticut

## Abstract

This cross-sectional study estimates US obesity prevalence using newly proposed anthropometric criteria.

## Introduction

Obesity has historically been defined using body mass index (BMI). However, BMI does not account for adipose tissue, limiting its accuracy. *The Lancet* Diabetes & Endocrinology Commission created a revised obesity definition including anthropometric measures (waist circumference [WC], waist-to-hip ratio [WHR], and waist-to-height ratio [WHtR]),^1^ encompassing and subcategorizing preclinical obesity (excess adiposity without organ dysfunction or physical impairment) and clinical obesity (a disease). Though more than 70 organizations have endorsed this definition,^1^ its use in practice has not been evaluated. Since obesity prevalence has implications for screening, risk assessment, and public health efforts, we estimated US obesity prevalence using these criteria.

## Methods

We analyzed adults with BMI (calculated as weight in kilograms divided by height in meters squared) measured in the 2017-2023 National Health and Nutrition Examination Survey (NHANES) (eFigure in Supplement 1).^2^ Obesity was defined using BMI, WC, WHR, and WHtR (eTables 1 and 2 in Supplement 1).^1^ We examined survey-weighted obesity prevalence and anthropometric criteria and compared subgroups using prevalence ratios (eMethods in Supplement 1). This study followed the STROBE guideline and was exempted from review and informed consent by the Yale institutional review board since NHANES data are deidentified.

## Results

The study included 14 414 participants representing 237 700 000 US adults. Survey-weighted obesity prevalence was 75.2% (95% CI, 73.8%-76.5%) overall, including 100% (95% CI, 99.9%-100%) among adults with BMI of 30 or greater, 80.4% (95% CI, 78.6%-82.1%) with BMI 25 to less than 30, and 38.5% (95% CI, 36.4%-40.6%) with BMI less than 25 (Figure). Prevalence was similar for men and women and higher for Hispanic adults and increased substantially with age (Table). Prevalence did not change from 2017-2020 to 2021-2023. The most common abnormal anthropometric criterion was WHtR above 0.50 (80.0% [95% CI, 78.6%-81.3%] of adults), followed by elevated WHR (73.1% [95% CI, 71.6%-74.6%]) and elevated WC (58.3% [95% CI, 56.8%-59.7%]), and 39.9% (95% CI, 38.0%-41.0%) of all adults had BMI of 30 or greater. Obesity prevalence using an alternative cutoff of WHtR above 0.60 was 58.4% (95% CI, 56.7%-60.2%) (Figure).

**Figure.**
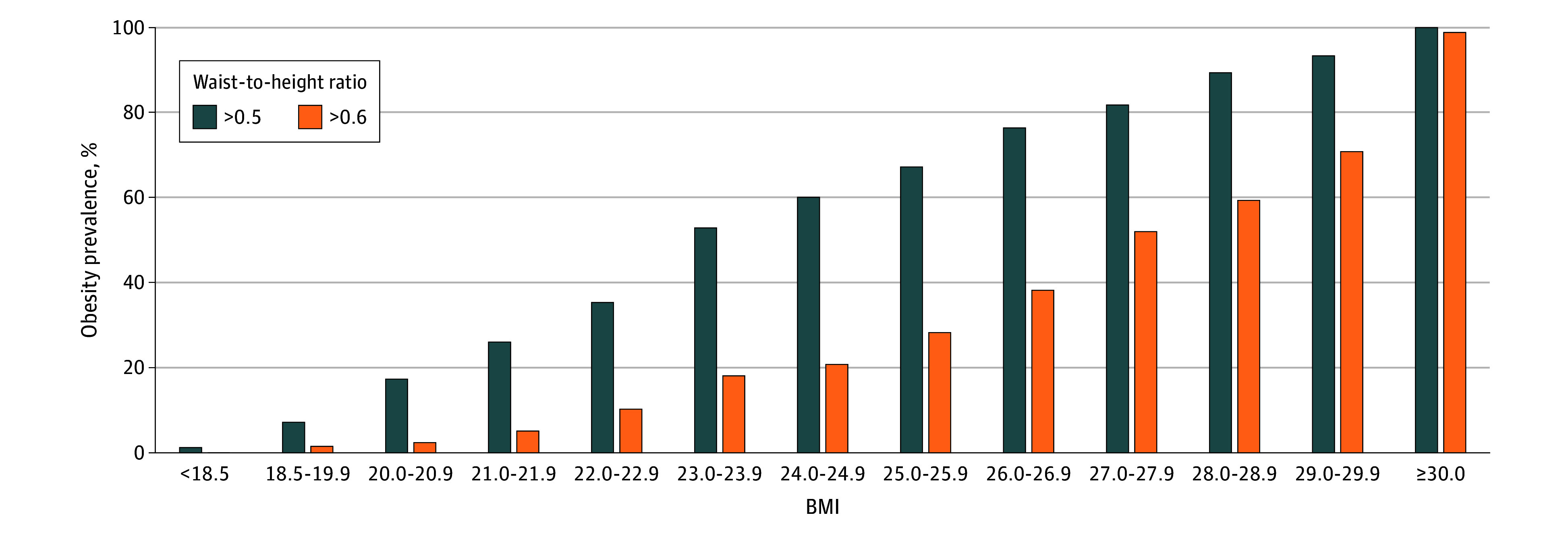
US Prevalence of Obesity Across Body Mass Index (BMI) Categories With Incorporation of Anthropometric Criteria Obesity definition and elevated anthropometric measures (waist circumference, waist-to-hip ratio, and waist-to-height ratio) were based on criteria cited by the 2025 *Lancet* Diabetes & Endocrinology Commission. Alternative obesity definitions were used for Asian adults (eTable 2 in Supplement 1). A waist-to-height ratio >0.5 was considered a criterion for obesity in the primary analysis and an alternative waist-to-height ratio >0.6 was also evaluated. All prevalence estimates were survey weighted and age standardized using data for US adults from the National Health and Nutrition Examination Survey. BMI was calculated as weight in kilograms divided by height in meters squared.

**Table.** US Prevalence of Obesity and Excess Adiposity Based on BMI and Anthropometric Criteria^a^

Characteristic	WHtR >0.50					WHtR >0.60			
	Prevalence, % (95% CI)				PR (95% CI)^b^	Prevalence, % (95% CI)			PR (95% CI)^b^
	Total	BMI <30 plus 2 anthropometric criteria	BMI 30 to <40 plus 1 anthropometric criterion	BMI ≥40		Total	BMI <30 plus 2 anthropometric criteria	BMI 30 to <40 plus 1 anthropometric criterion	
Total population (N = 14 414)	75.2 (73.8-76.5)	34.4 (33.4-35.4)	31.8 (30.3-33.2)	9.0 (8.2-10.0)	NA	58.4 (56.7-60.2)	18.0 (17.3-18.8)	31.4 (29.9-32.8)	NA
Sex									
Men	76.1 (74.4-77.8)	36.1 (34.2-37.9)	33.8 (31.5-36.1)	6.3 (5.4-7.3)	1.02 (0.99-1.06)	51.8 (49.3-54.3)	12.3 (11.3-13.5)	33.2 (30.9-35.6)	0.80 (0.76-0.84)
Women	74.3 (72.4-76.2)	33.0 (31.5-34.4)	29.7 (28.1-31.4)	11.7 (10.5-13.0)	1 [Reference]	64.7 (62.7-66.7)	23.5 (22.2-25.0)	29.5 (27.9-31.2)	1 [Reference]
Race and ethnicity									
Hispanic	81.4 (79.0-83.6)	38.1 (36.0-40.3)	35.5 (33.3-37.7)	7.8 (6.4-9.5)	1.10 (1.07-1.14)	61.5 (58.4-64.5)	18.7 (17.2-20.3)	35.0 (32.8-37.3)	1.08 (1.02-1.15)
Non-Hispanic Asian	70.1 (66.4-73.6)	42.1 (39.0-45.1)	27.0 (23.3-31.1)	1.0 (0.6-1.7)	0.95 (0.90-1.00)	59.4 (55.9-62.8)	31.8 (28.6-35.2)	26.6 (23.0-30.5)	1.05 (0.97-1.12)
Non-Hispanic Black	73.9 (72.2-75.6)	24.7 (23.2-26.4)	34.4 (32.5-36.4)	14.8 (13.3-16.4)	1.00 (0.97-1.03)	60.6 (58.1-63.1)	12.1 (11.0-13.3)	33.7 (31.8-35.7)	1.07 (1.02-1.12)
Non-Hispanic White	73.7 (71.9-75.5)	34.4 (32.7-36.1)	30.4 (28.3-32.6)	8.9 (7.8-10.2)	1 [Reference]	56.7 (54.3-59.1)	17.7 (16.5-18.8)	30.1 (28.0-32.3)	1 [Reference]
Other^c^	77.8 (73.5-81.5)	33.7 (28.5-39.2)	33.2 (27.7-39.3)	10.9 (7.8-15.0)	1.05 (1.00-1.11)	61.3 (56.2-66.2)	17.9 (13.5-23.3)	32.5 (27.2-38.4)	1.08 (0.99-1.17)
Total population by age, y									
18-29	48.4 (45.1-51.7)	16.4 (14.4-18.6)	25.0 (22.1-28.3)	6.9 (5.7-8.3)	1 [Reference]	37.3 (33.9-40.9)	6.3 (5.1-7.9)	24.0 (21.1-27.3)	1 [Reference]
30-39	72.3 (69.3-75.1)	28.8 (26.1-31.6)	31.6 (29.0-34.4)	11.9 (10.1-14.0)	1.49 (1.39-1.60)	55.4 (52.4-58.3)	12.3 (10.5-14.3)	31.2 (28.5-34.0)	1.49 (1.34-1.63)
40-49	80.9 (78.4-83.1)	35.0 (32.0-38.2)	34.6 (32.1-37.2)	11.2 (9.4-13.3)	1.67 (1.56-1.78)	62.6 (58.9-66.1)	17.0 (14.4-19.8)	34.4 (31.8-37.0)	1.68 (1.52-1.84)
50-59	87.0 (84.5-89.2)	39.6 (37.0-42.3)	35.6 (33.1-38.2)	11.8 (10.2-13.7)	1.80 (1.67-1.92)	68.0 (65.0-70.8)	20.7 (18.6-23.0)	35.5 (33.0-38.1)	1.82 (1.65-1.99)
60-69	90.1 (88.1-91.8)	46.7 (44.0-49.5)	35.7 (32.7-38.9)	7.6 (6.4-9.0)	1.86 (1.76-1.97)	69.5 (66.1-72.7)	26.3 (24.4-28.4)	35.6 (32.5-38.8)	1.86 (1.71-2.02)
70-79	92.3 (90.7-93.7)	50.2 (46.8-53.7)	36.1 (32.3-40.0)	6.0 (4.8-7.5)	1.91 (1.78-2.03)	75.3 (72.4-77.9)	33.3 (30.3-36.4)	36.0 (32.3-39.9)	2.02 (1.83-2.21)
≥80	92.0 (89.3-94.1)	62.5 (58.8-66.1)	26.0 (23.0-29.2)	3.5 (1.9-6.4)	1.90 (1.75-2.05)	71.5 (67.2-75.5)	42.0 (38.4-45.8)	26.0 (23.0-29.2)	1.92 (1.71-2.13)
Men by age, y									
18-29	45.4 (40.8-50.0)	14.0 (11.8-16.5)	26.7 (22.6-31.3)	4.7 (3.6-6.1)	1 [Reference]	32.4 (28.0-37.2)	2.5 (1.7-3.7)	25.2 (21.2-29.8)	1 [Reference]
30-39	74.1 (69.9-77.9)	31.6 (27.1-36.4)	34.1 (30.2-38.2)	8.4 (6.1-11.6)	1.63 (1.46-1.81)	49.0 (44.3-53.6)	7.1 (5.3-9.6)	33.4 (29.6-37.4)	1.51 (1.27-1.76)
40-49	83.0 (79.3-86.1)	35.0 (30.8-39.5)	39.4 (35.6-43.3)	8.5 (6.1-11.9)	1.83 (1.67-2.02)	58.4 (53.3-63.3)	10.8 (8.3-13.9)	39.1 (35.3-43.0)	1.80 (1.52-2.09)
50-59	90.7 (88.1-92.8)	43.9 (40.1-47.7)	37.9 (33.8-42.1)	8.9 (6.8-11.7)	2.00 (1.81-2.19)	60.0 (55.0-64.8)	13.3 (10.5-16.6)	37.8 (33.8-42.1)	1.85 (1.60-2.10)
60-69	92.4 (89.7-94.4)	51.6 (46.0-57.2)	36.0 (30.3-42.0)	4.8 (3.6-6.2)	2.04 (1.84-2.23)	60.8 (55.7-65.7)	20.2 (17.8-23.0)	35.8 (30.1-41.9)	1.88 (1.60-2.15)
70-79	93.5 (91.2-95.2)	52.7 (47.6-57.8)	37.3 (31.8-43.1)	3.5 (2.4-5.0)	2.06 (1.86-2.26)	67.2 (62.6-71.5)	26.5 (22.6-30.9)	37.2 (31.8-43.0)	2.07 (1.76-2.39)
≥80	94.6 (91.3-96.6)	68.5 (63.7-73.0)	24.9 (20.4-30.1)	1.1 (0.4-2.9)	2.08 (1.88-2.29)	61.1 (54.8-67.1)	35.1 (30.0-40.4)	24.9 (20.4-30.1)	1.89 (1.56-2.22)
Women by age, y									
18-29	51.7 (47.8-55.5)	19.2 (16.0-22.7)	23.2 (19.8-27.0)	9.3 (7.5-11.4)	1 [Reference]	42.6 (38.4-46.9)	10.6 (7.9-14.0)	22.7 (19.3-26.5)	1 [Reference]
30-39	70.5 (67.1-73.7)	26.0 (23.4-28.7)	29.0 (25.6-32.8)	15.5 (13.1-18.2)	1.37 (1.25-1.48)	61.9 (58.2-65.5)	17.5 (15.4-19.9)	28.9 (25.5-32.6)	1.45 (1.29-1.62)
40-49	78.7 (74.8-82.2)	35.0 (30.9-39.4)	29.7 (26.4-33.2)	14.0 (11.3-17.1)	1.52 (1.42-1.63)	67.0 (62.6-71.1)	23.4 (19.9-27.3)	29.6 (26.4-33.1)	1.57 (1.42-1.72)
50-59	83.6 (78.9-87.4)	35.6 (32.2-39.2)	33.5 (29.0-38.2)	14.5 (11.8-17.7)	1.62 (1.51-1.73)	75.5 (71.4-79.1)	27.6 (24.1-31.4)	33.4 (28.9-38.1)	1.77 (1.60-1.94)
60-69	88.0 (84.9-90.5)	42.3 (38.8-46.0)	35.5 (31.5-39.8)	10.2 (8.6-12.0)	1.70 (1.59-1.81)	77.4 (73.3-81.1)	31.9 (28.3-35.7)	35.4 (31.3-39.7)	1.82 (1.65-1.98)
70-79	91.4 (89.0-93.3)	48.2 (44.0-52.5)	35.1 (31.0-39.4)	8.1 (6.3-10.4)	1.77 (1.64-1.90)	82.0 (78.5-85.0)	38.9 (34.6-43.4)	35.0 (31.0-39.3)	1.93 (1.73-2.13)
≥80	90.4 (86.5-93.3)	58.6 (53.1-63.9)	26.7 (22.2-31.7)	5.1 (2.6-9.8)	1.75 (1.59-1.91)	78.4 (73.3-82.7)	46.6 (41.3-52.0)	26.7 (22.1-31.7)	1.84 (1.61-2.07)
Total population by BMI									
<25	36.9 (34.8-39.1)	36.9 (34.8-39.1)	NA	NA	1 [Reference]	10.3 (8.7-12.1)	10.3 (8.7-12.1)	NA	1 [Reference]
25 to <30	79.5 (77.5-81.2)	79.5 (77.5-81.2)	NA	NA	2.15 (2.02-2.28)	47.3 (45.4-49.2)	47.3 (45.4-49.2)	NA	4.61 (3.86-5.36)
30 to <40	100 (99.9-100)	NA	100 (99.9-100)	NA	2.71 (2.56-2.86)	98.7 (98.1-99.1)	NA	98.7 (98.1-99.1)	9.62 (8.09-11.15)

Abbreviations: BMI, body mass index (calculated as weight in kilograms divided by height in meters squared); NA, not applicable; PR, prevalence ratio; WC, waist circumference; WHR, waist-to-hip ratio; WHtR, waist-to-height ratio.

^a^ Obesity definitions and elevated anthropometric measures (WC, WHR, and WHtR) were based on criteria cited by the 2025 *Lancet* Diabetes & Endocrinology Commission. An alternative WHtR threshold of greater than 0.60 was also evaluated. Alternative obesity definitions and thresholds for BMI and WC were used for Asian adults (eTable 2 in Supplement 1).

^b^ Prevalence ratios represent the total prevalence of obesity using BMI and anthropometric criteria compared with the designated reference group within each category.

^c^ Includes all adults who self-reported as “other non-Hispanic race including non-Hispanic multiracial” on the National Health and Nutrition Examination Survey form.

## Discussion

This study, using a new obesity definition, estimated that 75.2% of US adults have obesity. In a study evaluating this definition using dual-energy X-ray absorptiometry (DEXA) and WC only, obesity prevalence (39%) was similar to prevalence using only BMI (40%).^3^ However, that study represented a smaller population and did not include WHR or WHtR. Using all 3 anthropometric criteria with BMI resulted in a substantial increase in estimated obesity prevalence vs using only BMI of 30 or greater.

We found that 100% of participants with BMI of 30 or greater had obesity, indicating practically all adults at this BMI likely have excess adiposity. Four in five adults with overweight and 38.5% with normal BMI also had obesity, reclassifying an additional 34% of the study population. Estimated prevalence rose substantially with age.

These findings demonstrate the impact of anthropometric thresholds, particularly since 80.0% of adults had WHtR above 0.5. Though this value was cited by the *Lancet* Commission and identifies cardiometabolic risk,^1,4,5^ the commission emphasized that additional research was required for this cutoff.^1^ Our study cannot determine optimal cutoffs or associations with health risks and confirms more evidence is needed before wider implementation. Since nearly all adults aged 50 years or older were classified as having obesity, age-specific thresholds are also needed. Nonetheless, using a conservative WHtR cutoff (>0.6) still resulted in higher obesity prevalence estimates, highlighting potential limitations of using BMI only.

Though we estimated prevalence of total obesity, the *Lancet* Commission separately defined preclinical and clinical obesity using additional classifications we could not evaluate. Our study also did not include DEXA, evaluate clinical outcomes, or assess indications for specific therapies.

This cross-sectional study estimated that 3 in 4 US adults had obesity based on a new definition. Further work is needed to evaluate and adapt this definition for research, screening, management, public health, and policy decisions. Additional investigation on anthropometric criteria is needed to ensure definitions are appropriate.

## Notes

Supplement 1. **eMethods.** Study Cohort, Definitions, and Statistical Analysis **eTable 1.** NHANES Variables Used for Study Definitions **eTable 2.** Criteria for Obesity Based on Values Cited by the *Lancet* Commission **eFigure.** Study Cohort Selection
eReferences

Supplement 2.
Data Sharing Statement
