# Supplementary material for: Body Mass Index and Anthropometric Criteria to Assess Obesity
Source: JAMA Netw Open. 2025 Dec 29;8(12):e2549124. doi: 10.1001/jamanetworkopen.2025.49124 (PMC12750250; doi:10.1001/jamanetworkopen.2025.49124)
Supplement: Supplement 1. — eMethods. Study Cohort, Definitions, and Statistical Analysis eTable 1. NHANES Variables Used for Study Definitions eTable 2. Criteria for Obesity Based on Values Cited by the Lancet Commission eFigure. Study Cohort Selection eReferences [file jamanetwopen-e2549124-s001.pdf]

Supplementary Online Content

Al-Roub NM, Malik D, Essa M, et al. Body mass index and anthropometric criteria to assess obesity. *JAMA Netw Open*. 2025;8(12):e2549124.
doi:10.1001/jamanetworkopen.2025.49124

eMethods. Study Cohort, Definitions, and Statistical Analysis
eTable 1. NHANES Variables Used for Study Definitions
eTable 2. Criteria for Obesity Based on Values Cited by the *Lancet* Commission
eFigure. Study Cohort Selection
eReferences

This supplementary material has been provided by the authors to give readers additional information about their work.

© 2025 Al-Roub NM et al. *JAMA Network Open*.

eMethods. Study Cohort, Definitions, and Statistical Analysis

Study Cohort & Definitions

The National Health and Nutrition Examination Survey (NHANES) is a nationally representative survey of the U.S. population.[1] Hip circumference was collected beginning in 2017, and therefore only the 2017-2020 and 2021-2023 survey cycles were used. Adults ≥18 years of age who were pregnant or who could not otherwise be classified using cited anthropometric criteria were excluded (**eFigure 1**). All demographic data were self-reported, including race/ethnicity designated in the NHANES survey (non-Hispanic white, non-Hispanic black, non-Hispanic Asian, Hispanic or other race/ethnicity). Obesity was defined using body mass index (BMI) and anthropometric measures (waist circumference [WC], waist-to-hip ratio [WHR], and waist-to-height ratio [WHtR]) based on criteria and elevated values cited by the *Lancet* Diabetes and Endocrinology Commission (**eTables 1 & 2**). Since WHtR >0.50 was specifically cited,[2] this was used as the provisional criterion in our primary analyses. In exploratory analyses, we used WHtR >0.60 as a previously proposed cutoff for high central adiposity.[3] A prior study has also proposed alternative WHtR cutoffs ranging from 0.55-0.59 for different demographic groups based on visceral adipose tissue identified with DEXA,[4] and so the >0.60 threshold was also used as a potential upper bound for defining elevated WHtR in our study. Since we could not separately classify preclinical and clinical obesity based on the *Lancet* definition, all prevalence estimates for obesity represent the combined prevalence of preclinical and clinical obesity.

Survey Design and Weighting

All analyses accounted for the complex, multistage probability sampling design of NHANES. Sampling weights incorporated adjustments for unequal probability of selection,

© 2025 Al-Roub NM et al. *JAMA Network Open*.

survey non-response, and post-stratification to match the demographic structure of the U.S. population, in accordance with National Center for Health Statistics (NCHS) analytic guidelines. Clustering and stratification variables were applied to account for the survey design, and all variance estimates were calculated using Taylor series linearization, as recommended by the NCHS. Prevalence estimates were reported as weighted percentages with corresponding 95% confidence intervals (CIs). Statistical significance was defined as a two-sided P-value less than 0.05. All analyses were conducted using Stata BE version 18 (StataCorp, 2023).

Age Standardization

To adjust for differences in age distributions across subgroups and survey cycles, direct standardization was performed. Survey-weighted, age-specific proportions of the outcome were first estimated within each age group. These estimates were then standardized to the 2020 U.S. Census population. This procedure was implemented using Stata's stdize() function within the survey-weighted estimation framework, ensuring that both NHANES survey weights and age-standardization weights were appropriately applied. By combining survey weights and age-standardization weights, the standardized prevalence estimates reflect both the complex sampling design and the age distribution of the U.S. population.

Prevalence Ratios

Prevalence ratios were estimated using Poisson regression models with survey-weighted estimation incorporating sampling weights, clustering, and stratification. All variance estimates from the Poisson models were calculated using Taylor series linearization to account for the complex sampling design and intra-cluster correlation. All models evaluating trends across survey cycles were adjusted for age, sex, and race/ethnicity. Model diagnostics confirmed that the assumption of no overdispersion was met.

© 2025 Al-Roub NM et al. *JAMA Network Open*.

To assess for changes in total obesity prevalence following the COVID-19 pandemic, prevalence ratios were similarly determined from Poisson regression comparing prevalence in the 2021-2023 survey to prevalence in the 2017-March 2020 survey, which served as the reference. These models were fully adjusted for age, sex, and race/ethnicity to account for demographic differences across time periods.

© 2025 Al-Roub NM et al. *JAMA Network Open*.

eTable 1. NHANES Variables Used for Study Definitions*

Variable	Definition
Body Mass Index (BMI)	Weight in kilograms divided by height in meters squared
Weight Circumference (WC)	Waist circumference in cm using a tape measure at the uppermost lateral border of the hip crest (ilium)[1]
Hip Circumference	Hip circumference in cm at the level of the maximum extension of the buttocks[1]
Waist to Hip Ratio (WHR)	Waist Circumference (cm) divided by Hip Circumference (cm)
Waist to Height Ratio (WHtR)	Waist Circumference (cm) divided by Height Circumference (cm)

**National Center for Health Statistics.* NHANES 2022 Interviewer Procedures Manual*. U.S. Department of Health and Human Services, Centers for Disease Control and Prevention (CDC).* 2022. *https://wwwn.cdc.gov/nchs/data/nhanes/public/2021/manuals/2022-Interviewer-Procedures-508.pdf*

© 2025 Al-Roub NM et al. *JAMA Network Open*.

eTable 2. Criteria for Obesity Based on Values Cited by the *Lancet* Commission

Men (excluding Asian adults)	Women (excluding Asian adults)
BMI ≥40 kg/m^2	BMI ≥40 kg/m^2
BMI 30-39.9 kg/m^2 and ≥1 of the following:<br>*Waist Circumference >102 cm*<br>*Waist-Hip Ratio >0.90*<br>*Waist-Height Ratio >0.50*	BMI 30-39.9 kg/m^2 and ≥1 of the following:<br>*Waist Circumference >88 cm*<br>*Waist-Hip Ratio >0.85*<br>*Waist-Height Ratio >0.50*
BMI <30 kg/m^2 and ≥2 of the following:<br>*Waist Circumference >102 cm*<br>*Waist-Hip Ratio >0.90*<br>*Waist-Height Ratio >0.50*	BMI <30 kg/m^2 and ≥2 of the following:<br>*Waist Circumference >88 cm*<br>*Waist-Hip Ratio >0.85*<br>*Waist-Height Ratio >0.50*
**Asian Men**	**Asian Women**
BMI ≥40 kg/m^2	BMI ≥40 kg/m^2
BMI 27.5-39.9 kg/m^2 and ≥1 of the following:<br>*Waist Circumference >90 cm*<br>*Waist-Hip Ratio >0.90*<br>*Waist-Height Ratio >0.50*	BMI 27.5-39.9 kg/m^2 and ≥1 of the following:<br>*Waist Circumference >80 cm*<br>*Waist-Hip Ratio >0.85*<br>*Waist-Height Ratio >0.50*
BMI <27.5 kg/m^2 and ≥2 of the following:<br>*Waist Circumference >90 cm*<br>*Waist-Hip Ratio >0.90*<br>*Waist-Height Ratio >0.50*	BMI <27.5 kg/m^2 and ≥2 of the following:<br>*Waist Circumference >80 cm*<br>*Waist-Hip Ratio >0.85*<br>*Waist-Height Ratio >0.50*

© 2025 Al-Roub NM et al. *JAMA Network Open*.

eFigure. Study Cohort Selection

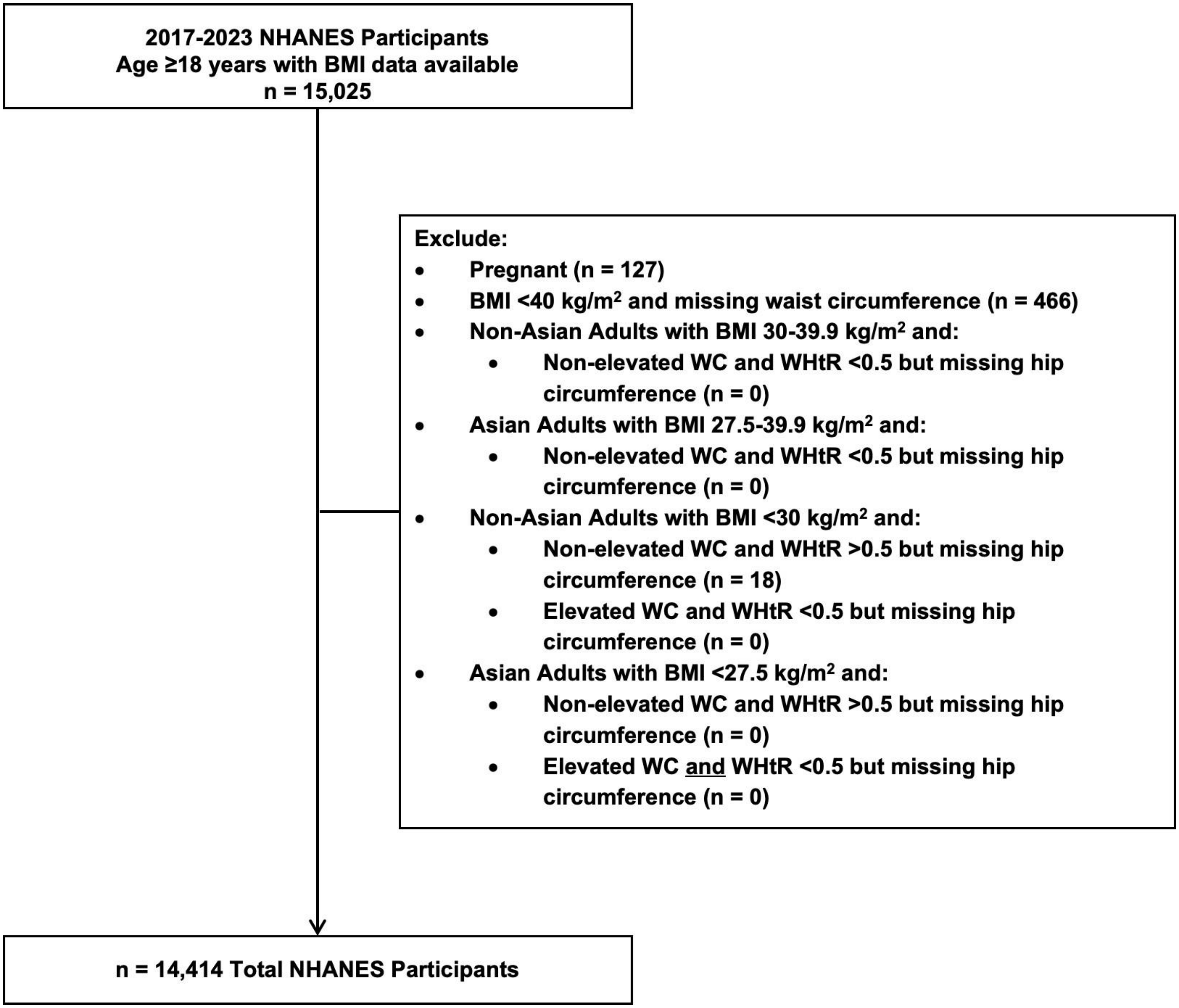

As shown on Figure, pregnant adults and participants with missing data who could not otherwise be classified using *Lancet* definitions of obesity were excluded. In analyses using WHtR >0.60, there were n = 0 adults missing hip circumference who could not otherwise be classified as having obesity or not (total n = 14,432). BMI = body mass index; WC = waist circumference; WHtR = Waist-to-height ratio.

© 2025 Al-Roub NM et al. *JAMA Network Open*.

eReferences:

1. U.S. Centers for Disease Control and Prevention. US National Health and Nutrition Examination Survey. Accessed February 4, 2025. http://www.cdc.gov/nchs/nhanes.htm
2. Rubino F, Cummings DE, Eckel RH, Cohen RV, Wilding JPH, Brown WA, et al. Definition and diagnostic criteria of clinical obesity. Lancet Diabetes Endocrinol. 2025 Mar;13(3):221–62.
3. National Institute for Health and Care Excellence. Overweight and Obesity Management: NICE Guideline. Accessed March 5, 2025. https://www.nice.org.uk/guidance/ng246/chapter/Identifying-and-assessing-overweight-obesity-and-central-adiposity
4. Itani L, El Ghoch M. Waist-to-Height Ratio Cut-Off Points for Central Obesity in Individuals with Overweight Across Different Ethnic Groups in NHANES 2011-2018. Nutrients. 2024 Nov 8;16(22):3838.

© 2025 Al-Roub NM et al. *JAMA Network Open*.
