# Supplementary material for: Body Mass Index and Anthropometric Criteria to Assess Obesity
Source: JAMA Netw Open. 2025 Dec 29;8(12):e2549124. doi: 10.1001/jamanetworkopen.2025.49124 (PMC12750250; doi:10.1001/jamanetworkopen.2025.49124)
Supplement: Supplement 2. — Data Sharing Statement [file jamanetwopen-e2549124-s002.pdf]

Data Sharing Statement

Al-Roub. Body Mass Index and Anthropometric Criteria to Assess Obesity. *JAMA Netw Open*. Published December 29, 2025. doi:10.1001/jamanetworkopen.2025.49124

Data
Data available: Yes
Data types: Deidentified participant data
How to access data: All participant data are available at https://www.cdc.gov/nchs/nhanes/index.html
When available: With publication

Supporting Documents
Document types: Other (please specify)
Additional Information: All participant data are available at https://www.cdc.gov/nchs/nhanes/index.html
How to access documents: All participant data are available at https://www.cdc.gov/nchs/nhanes/index.html
When available: With publication

Additional Information
Who can access the data: All participant data are available at https://www.cdc.gov/nchs/nhanes/index.html
Types of analyses: All participant data are available at https://www.cdc.gov/nchs/nhanes/index.html
Mechanisms of data availability: All participant data are available at https://www.cdc.gov/nchs/nhanes/index.html
